# Planar Pressure Field Determination in the Initial Merging Zone of an Annular Swirling Jet Based on Stereo-PIV Measurements

**DOI:** 10.3390/s8127596

**Published:** 2008-11-28

**Authors:** Maarten Vanierschot, Eric Van den Bulck

**Affiliations:** 1 Department of Mechanical Engineering, Katholieke Universiteit Leuven, Celestijnenlaan 300A, B-3001 Heverlee, Belgium

**Keywords:** indirect pressure measurement, swirling flow, Poisson's equation

## Abstract

In this paper the static pressure field of an annular swirling jet is measured indirectly using stereo-PIV measurements. The pressure field is obtained from numerically solving the Poisson equation, taken into account the axisymmetry of the flow At the boundaries no assumptions are made and the exact boundary conditions are applied. Since all source terms can be measured using stereo-PIV and the boundary conditions are exact, no assumptions other than axisymmetry had to be made in the calculation of the pressure field. The advantage of this method of indirect pressure measurement is its high spatial resolution compared to the traditional pitot probes. Moreover this method is non-intrusive while the insertion of a pitot tube disturbs the flow It is shown that the annular swirling flow can be divided into three regimes: a low, an intermediate and a high swirling regime. The pressure field of the low swirling regime is the superposition of the pressure field of the non-swirling jet and a swirl induced pressure field due to the centrifugal forces of the rotating jet. As the swirl increases, the swirl induced pressure field becomes dominant and for the intermediate and high swirling regimes, the simple radial equilibrium equation holds.

## 1. Introduction

The most widely used direct method to measure the static pressure in a flow field is by insertion of a Pitot tube. However this method has some disadvantages. It disturbs the flow field and the probe dimensions are in the order of a few mm. In order to obtain sufficient spatial resolution, the flow field dimensions have to be very large compared to the probe dimensions. Since pressure and velocity are linked by the Navier-Stokes equations, the static pressure can also be measured indirectly by measuring the velocity field. The Navier-Stokes equations form a set of non-linear partial differential equations, consisting of a continuity equation, three momentum equations and an energy equation. The momentum equations are the link between the static pressure in the flow field and the velocity field. There exist 2 strategies to measure the pressure field indirectly. The first strategy is direct spatial integration of the momentum equations [1, 2]. The pressure gradients are integrated directly, starting from reference points in the flow field. In these reference points the pressure is known (Dirichlet boundary conditions) and they are usually situated in the outer part of the flow field. The second strategy is solving a Poisson equation for the pressure field [2, 3]. By taking the divergence of the momentum equations they can be rewritten as a Poisson equation, with the source terms known from measurements. The pressure field is then computed by solving the equation using the appropriate boundary conditions on each boundary of the domain (Neumann or Dirichlet conditions).

In this paper a study is made of the static pressure field of a swirling annular jet. Three components of velocity in a plane through the central axis are measured using stereoscopic PIV measurements. The pressure field is computed by solving the Poisson equation using exact boundary conditions based on the momentum equations. The mean flow field is found to be axisymmetric and hence all the source terms in the Poisson equation could be determined. This approach differs from previous studies found in literature which could not measure all source terms and additional assumptions had to be made for the unknown values or for the boundary conditions. Four different swirl cases were investigated: a non-swirling, a low, an intermediate and a high swirling jet. For a low swirling jet, the static pressure field is the superposition of the pressure field of a non-swirling jet and a swirl induced pressure field. This swirl induced pressure field originates as a balance of the centrifugal forces due to the rotating jet. As the swirl is increased, it's induced pressure field dominates and the radial momentum equation can be simplified to the simple radial equilibrium equation, which is a balance between pressure gradients in the radial direction and centrifugal forces.

## 2. Experimental procedure and flow measurement

### 2.1. Experimental setup

**Figure 1.**
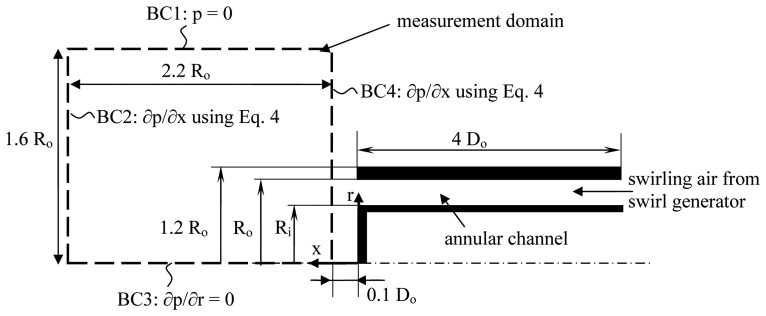
Schematic view of the experimental configuration, the measurement domain (dashed line) and the boundary conditions for solving the Poisson equation for pressure.

A schematic view of the experimental setup is shown in Fig. 1. Swirling air comes from a moveable block swirl generator [4] and enters an annular channel with outer radius *R_o_* = 13.5 mm, inner radius *R_i_* = 0.65 *R_o_* and length 4 *D_o_*. The area blockage ratio *B* of the jet, defined as the ratio between the inner and outer surface of the annular channel or $B=R_{i}^{2}/R_{o}^{2}$, is 0.42. The channel expands into the quiescent surroundings (free jet). More details concerning the air supply and swirl generator can be found in the study of Vanierschot et al. [5]. The *x*-axis of the cylindrical polar coordinate system (*x*, *r*, *θ*) is coincident with the central axis of the annulus and the origin is located at the exit of the channel. The Reynolds number is 15500 and is based on the mean axial velocity *U_0_*, the hydraulic diameter of the annular channel, *D_o_* − *D_i_*, and the viscosity of air at 20°C, *ν* = 15.1 mm^2^/s. The dimensionless swirl number *S*, first proposed by Beér et al. [6], expresses the amount of rotation of the flow It is defined as

(1) $$S=\frac{\int_{R_{i}}^{R_{o}}\text{UW}r^{2}\text{dr}}{R_{o}\int_{R_{i}}^{R_{o}}U^{2}\text{rdr}},$$

with *U* and *W* the local mean velocities in the axial and azimuthal direction respectively. In this paper, a total of 4 different swirl numbers *S* are investigated: one at zero swirl, one at low swirl (*S* = 0.18), one at intermediate swirl (*S* = 0.37) and one at high swirl (*S* = 0.74).

### 2.2. Velocity measurement technique

**Figure 2.**
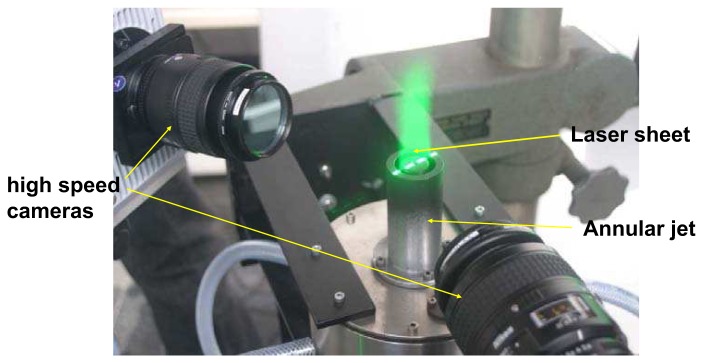
Photo of the experimental configuration.

The flow field is measured using the stereoscopic particle image velocimetry (PIV) technique. A photo of the experimental configuration is shown in Fig. 2. A Dual Cavity Nd:YLF Pegasus-PIV laser from NewWave, with a wavelength of 527 nm and a pulse energy of 10mJ @ 1000Hz, generates a green light sheet perpendicular to the exit of the annular channel. The sheet lies in a (xr)-plane through the central axis. This allows for the simultaneous measurement of the axial (U), radial (V) and azimuthal (W) velocity components. The flow is seeded with small droplets of oil (diameters between 0.2 and 1 *μ*m), generated by a Palas type AGF 10.0 Liquid Nebulizer. The separation time between the two pulses, 15 *μ*s, is chosen as large as possible fulfilling the conditions that the maximum out-of-plane displacement is less than 1/4 of the light sheet thickness and the maximum in-plane pixel displacement is within 1/4 of the interrogation window size [7]. The PIV images are taken using 2 ′HighSpeedStar 5′ CMOS cameras, with a resolution of 1024 × 1024 px, mounted in the forward scatter direction under an angle of about 45 degrees with the laser sheet plane. The dimensions of the measurement plane are shown in Fig. 1. The recording technique used is ‘double frame / double exposure’ meaning that the 2 light pulses are recorded on 2 different images. The correlation peak is determined using cross-correlation of these two images. The calculation of the velocity vectors is done using the DaVis 7.1 Software of LaVision GmbH. This software is used only for this purpose and all the other post-processing is done using in-house developed software. In order to obtain a good spatial accuracy and a minimization of spurious data, the velocity vectors are calculated iteratively using window deformation. The process starts at an interrogation area of 32×32 pixels and after 5 iterations convergence is reached with a final interrogation area of 12×12 pixels with 50% overlap. The amount of spurious vectors is well below 1%. They are detected and removed by a median test as first proposed by Westerweel [8]. Calibration of the PIV system is done by means of fitting a camera pinhole model to the two cameras using a 3D calibration plate [9]. The obtained scale factor of the images yields a distance of 0.5 mm between two neighboring vectors in both streamwise and transverse direction. Compared to the typical dimensions of a pitot tube, this is a factor of four smaller in spatial resolution.

When calculating time averaged properties of a flow, an error is introduced by computing statistical quantities from a finite number of data samples. In this study *N* = 1024 measurements are taken for each swirl case. The acquisition frequency of the measurements is 125 Hz in order to obtain statistically independent samples. Using the theory of signal analysis, as described in Bendat et al. [10], the relative errors on the mean velocities are around 2% and the errors on the rms and shear stresses 4.3% and 6.1% respectively in the entire measurement domain. More details concerning the measurement errors and a validation of the PIV measurements can be found in the study of Vanierschot et al. [5].

## 3. Indirect pressure measurement using stereoscopic PIV data

### 3.1. Equations of motion

The Navier-Stokes equations who describe the motion of an incompressible, newtonian fluid are given by the continuity equation,

(2) ∇⋅U=0,

and the momentum equations

(3) $$\frac{\partial \text{U}}{\partial t}+\text{U}\cdot \nabla \text{U}=-\frac{1}{\rho }\nabla p+v\nabla^{2}\text{U},$$

with **U** the velocity vector, *ρ* the density and *ν* the kinematic viscosity of the fluid and *p* the static pressure. For turbulent flows, the velocity field can be decomposed into a mean flow U̅ and a fluctuating component **u**. For the annular jet flow in this study, the viscous terms in Eq. 3 can be neglected. As confirmed by the measurements, these terms are several orders of magnitude smaller than the other ones. Filling into Eq. 3 and taking the time average of the momentum equations results into the following expression in cylindrical coordinates (the bars are left out for the time-averaged quantities)

(4) $$-\frac{1}{\rho }\frac{\partial P}{\partial x}=U\frac{\partial U}{\partial x}+V\frac{\partial U}{\partial r}+\frac{\partial \bar{\text{uu}}}{\partial x}+\frac{1}{r}\frac{\partial r\bar{\text{uv}}}{\partial r}$$

for the momentum equation in the axial direction and

(5) $$-\frac{1}{\rho }\frac{\partial P}{\partial r}=U\frac{\partial V}{\partial x}+V\frac{\partial V}{\partial r}+\frac{\partial \bar{\text{uv}}}{\partial x}+\frac{1}{r}\frac{\partial r\bar{\text{vv}}}{\partial r}-\frac{W^{2}}{r}-\frac{\bar{ww}}{r}$$

for the momentum equation in the radial direction. In these expressions U, V and W are the mean velocities in the axial (x), the radial (r) and the azimuthal (*θ*) direction. The terms $\bar{uu}$, $\bar{\text{uv}}$, $\bar{\text{vv}}$ and $\bar{\text{ww}}$ arise from the time averaging of the turbulent fluctuations and are called Reynolds stresses.

The above momentum equations in the axial and radial direction describe a relation between the pressure field and the velocity field. By taking the divergence of Eqs. 4 and 5 and taking into account the continuity equation, one obtains an expression for the Laplacian of the pressure field for an axisymmetric flow as

(6) $$-\frac{1}{\rho }\nabla^{2}P={\left(\frac{\partial U}{\partial x}\right)}^{2}+2\frac{\partial U}{\partial r}\frac{\partial V}{\partial x}+{\left(\frac{\partial V}{\partial r}\right)}^{2}+\frac{V^{2}}{r^{2}}+\frac{\partial^{2}\bar{\text{uu}}}{\partial x^{2}}+\frac{2}{r}\frac{\partial^{2}r\bar{\text{uv}}}{\partial r\partial x}+\frac{1}{r}\frac{\partial^{2}r\bar{\text{vv}}}{\partial r^{2}}-\frac{1}{r}\frac{\partial }{\partial r}(W^{2}+\bar{\text{ww}}),$$

where ∇^2^*P* in cylindrical coordinates is given by

(7) $$\nabla^{2}P=\frac{\partial^{2}P}{\partial x^{2}}+\frac{1}{r}\frac{\partial }{\partial r}\left(r\frac{\partial P}{\partial r}\right).$$

The assumption of axisymmetry is validated as the measurements confirm the velocity profiles are symmetric within measurement accuracy for all swirl cases. Equation 6 expresses the relation between the time averaged pressure field and the time averaged velocity field and the Reynolds stresses. Since stereo-PIV measures the three velocity components at the same time, all terms on the RHS of Eq. 6 can be determined. Using the propagation of errors, the measurement uncertainty on the RHS of Eq. 6 is estimated to be around 4%. In order to solve the Poisson equation, a boundary condition needs to be specified for each boundary of the measurement domain. An overview of the applied boundary conditions is shown in Fig. 1. The first boundary lies in the quiescent environment at *r*/*R_o_* = 1.6. In this region, the pressure is very close to the environmental pressure [11]. Equation 6 calculates the pressure relatively to an arbitrary value, so if one wants to calculate the pressure difference with the surroundings, the pressure on this boundary is taken to be zero. For boundary two and four the pressure is not known and a Neumann boundary condition is applied. On these boundaries *∂p*/*∂x* can be calculated, based on Eq. 4. On boundary three (the central axis) a symmetry condition is applied in the radial direction, given *∂p*/*∂r* = 0.

### 3.2. Discretisation schemes

The velocity is measured in *N_p_* = 2183 equidistant points, 37 in the radial and 59 in the axial direction. To determine the static pressure *p*_*i*,*j*_ in an internal point (point not on a boundary of the measurement domain) with coordinate *i* in the axial and *j* in the radial direction, the first and second order gradients in Eq. 6 need to be calculated. These gradients are approximated by a second order central difference scheme. The first and second order derivatives of the pressure *p*_*i*,*j*_ are calculated as

(8) $$\begin{array}{ccc} {\frac{\partial p}{\partial x}|}_{i,j} & \approx & \frac{p_{i+1,j}-p_{i-1,j}}{2\Delta x} \end{array},$$

(9) $$\begin{array}{ccc} {\frac{\partial^{2}p}{\partial x^{2}}|}_{i,j} & \approx & \frac{p_{i+1,j}-2p_{i,j}+p_{i-1,j}}{{\left(\Delta x\right)}^{2}} \end{array},$$

where Δ*x* is the axial distance between two measurement points. A similar expression can be found for the gradients in the radial direction *∂p*/*∂r* and *∂^2^p*/*∂r^2^*.

The boundary condition for boundary 1 gives the equation *p*_*i*,*j*_ = 0 (Dirichlet condition). On boundary two, Eq. 4 is applied (Neumann condition). The LHS on boundary 2 is calculated using a second order upwind discretisation scheme which yields:

(10) $$\begin{array}{ccc} {\frac{\partial p}{\partial x}|}_{i,j} & \approx & \frac{3p_{i}-4p_{i-1,j}+p_{i-2,j}}{2\Delta x} \end{array}.$$

For the central axis on boundary 3 the partial derivative of the pressure is discretised using a second order upwind scheme giving for a symmetry boundary condition

(11) $$\begin{array}{ccc} {\frac{\partial p}{\partial r}|}_{i,j} & \approx & \frac{-p_{i,j+2}+4p_{i,j+1}-3p_{i,j}}{2\Delta r} \end{array}=0.$$

Finally, the LHS of Eq. 4 on boundary 4 is calculated using a second order upwind scheme as

(12) $$\begin{array}{ccc} {\frac{\partial p}{\partial x}|}_{i,j} & \approx & \frac{-p_{i+2,j}+4p_{i+1,j}-3p_{i,j}}{2\Delta x} \end{array}.$$

Using a grid study in combination with Richardson extrapolation, the discretisation error is estimated to be around 5%. The discretisation of Eq. 6 in combination with the boundary conditions leads to a set of algebraic equations with the pressure in the measurement points as the unknowns. This set of equations can be written in matrix notation as C· 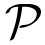 = S, where C (*N_p_* × *N_p_*) is the coefficient matrix, 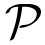 (*N_p_* × 1) is a vector with the unknown pressures and S (*N_p_* × 1) a vector with the source terms of the poisson equation or the RHS of the boundary conditions. The unknown pressures can then be determined by multiplying the matrix inversion of C with S.

## 4. Results and discussion

### 4.1. Non swirling jet, S=0

**Figure 3.**
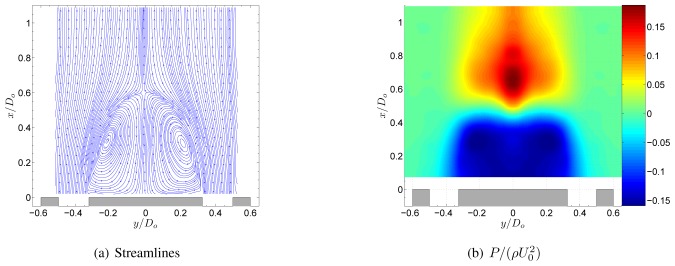
Flowfield of the annular jet at *S* = 0.

**Figure 4.**
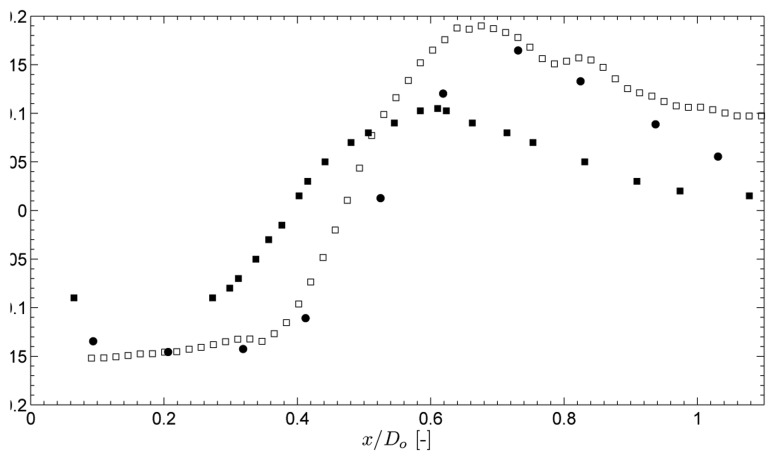
Normalised static pressure profile along the central axis. ■: data from Ko et al. [11], ●: data from Chigier et al. [12], □: data from this study.

The flow field of the non-swirling jet is shown in Fig. 3. The central tube of the jet acts as a bluff body to the flow, creating a central recirculation zone (CRZ) behind it. As shown in Fig. 4(a), this CRZ is an axisymmetric standing vortex with two centers. Downstream the CRZ closes at the stagnation point on the central axis at *x*/*D_o_* ≈ 0.6. The axial velocity profile behind the central tube is wake like with a static pressure inside the CRZ which is sub-atmospheric. Figure 4(b) shows the normalised static pressure $P/\left(\rho U_{0}^{2})\right)$. A local pressure minimum is situated at the centers of the CRZ. Local pressure minima that exist at the center of vortices can be used as a vortex identification criterion [13]. Near the stagnation point a pressure maximum is situated due to the decrease of velocity at the stagnation point according to the law of Bernouilli. Further downstream the pressure evolves towards atmospheric conditions. The non-swirling annular jet is a well known flow case in literature [5, 11, 12]. Figure 4 shows the normalised static pressure profile at the central axis of the geometry. This profile depends on the inlet velocity profile and the area blockage ratio *B* of the jet. For the geometry in this study *B* = 0.42. The obtained pressure profile agrees very well with the one in the study of Chigier et al. for which *B* was 0.43 [12]. The small differences are most likely attributed to the different inlet velocity profiles in both studies. The study of Ko et al. differs significantly The difference is attributed to the value of *B* = 0.20 in their study.

### 4.2. Low swirling jet, S=0.18

**Figure 5.**
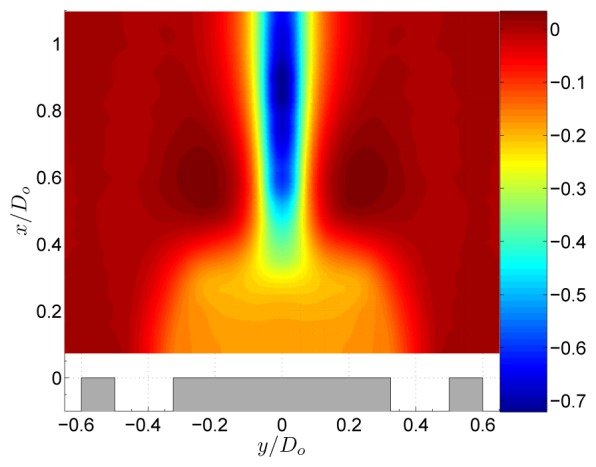
Normalised static pressure $P/\left(\rho U_{0}^{2}\right)$ of the annular jet at *S* = 0.18.

The pressure field of the swirling jet with swirl number *S* = 0.18 is shown in Fig. 5. At the central axis a large region of sub-atmospheric pressure originates from a balance of the centrifugal forces by radial pressure gradients. The centrifugal forces are described by the term *ρW^2^*/*r* in the radial momentum equation, Eq. 5. Downstream of the CRZ the streamlines are turned towards the central axis (Fig. 4(a)) and little downstream of the stagnation point, the jet streamlines reach their minimum radius. Since the conservation of angular momentum states that *rW* is constant along a streamline, a region of high azimuthal velocity exists immediately downstream of the stagnation point [5]. The high tangential velocities at low radius create large pressure gradients in the radial direction which yields the region of low pressure in Fig. 5.

To study the influence of swirl on the static pressure field, let us now consider a decomposition of the pressure field into a swirl induced component *P_S_* and a non-swirl component *P*_0_ as *P* = *P_S_* + *P*_0_. Filling this decomposition into Eq. 6 results into an expression for *P_S_* as

(13) $$\frac{1}{\rho }\nabla^{2}Ps=\frac{1}{r}\frac{\partial W^{2}}{\partial r}.$$

**Figure 6.**
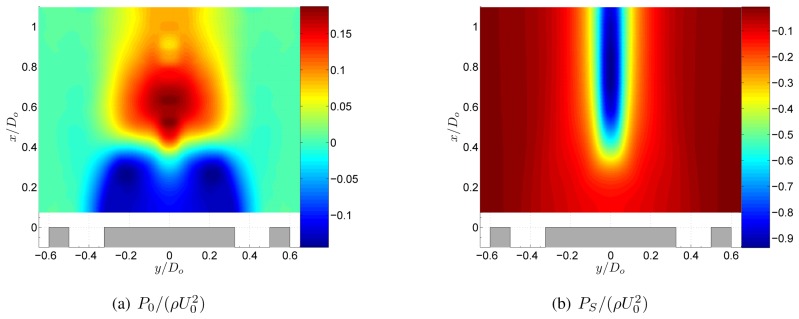
Decomposition of the pressure field of the annular jet at *S* = 0.18.

**Figure 7.**
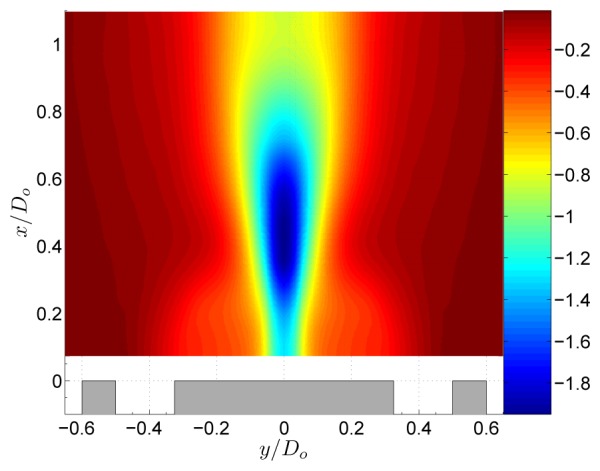
Normalised static pressure $P/\left(\rho U_{0}^{2}\right)$ of the annular jet at *S* = 0.37.

Both pressure fields are shown in Fig. 6. Comparing Fig. 5 with Fig. 6 shows that near the nozzle *P*_0_ is dominant. Further downstream, *x*/*D_o_* > 0.5, *P_S_* is dominant and *P*_0_ is negligible. Comparing *P*_0_ in Fig. 7(a) with *P* of the non-swirling jet in Fig. 4(b) shows that both pressure fields are very similar. The mean relative error between the two pressure fields, defined as $\bar{\left(P_{0}-P\right)}/\bar{P}$, is 2%. In other words, the static pressure field of a low swirling annular jet without the swirl induced pressure term in the poisson equation is approximately the same as the pressure field of the corresponding non-swirling jet. This was also shown in a study by Vanierschot et al. [5]. Hence the pressure field of a low swirling jet is the linear superposition of the pressure field of a non-swirling jet and a swirl induced pressure field.

### 4.3. Intermediate swirling jet, S=0.37

**Figure 8.**
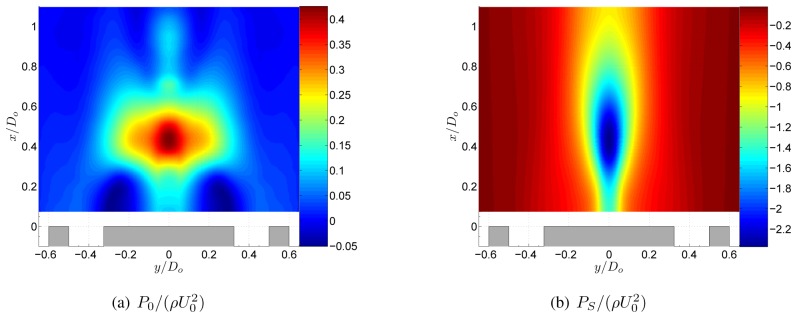
Decomposition of the pressure field of the annular jet at *S* = 0.37.

As the swirl is further increased, the static pressure in the low pressure region decreases as the azimuthal velocities increase (Fig. 7). The centrifugal forces create large pressure gradients near the central axis. Moreover the decrease of azimuthal velocity downstream yields to a positive pressure gradient in the axial direction. When this pressure gradient is sufficiently large, a region of recirculation along the central axis appears, called vortex breakdown. A comprehensive review on vortex breakdown can be found in the review article of Lucca-Negro and O'Doherty [14]. The decomposition of the pressure field in Fig. 8 shows that *P_S_* is dominant in the entire flow field and *P*_0_ is much smaller. As a result, Eq. 5 can be simplified to

(14) $$\frac{\partial P}{\partial r}=\rho \frac{W^{2}}{r}.$$

This equation is called the simplified radial equilibrium equation as it is the balance between pressure gradients and centrifugal forces [15].

### 4.4. High swirling jet, S=0.74

**Figure 10.**
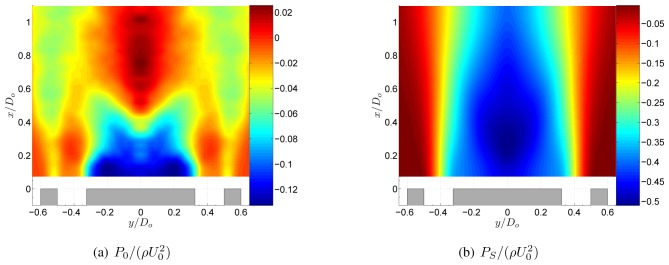
Decomposition of the pressure field of the annular jet at *S* = 0.74.

As the swirl is further increased, the centrifugal forces increase the radial expansion of the jet. The vortex breakdown bubble grows as the region of recirculation along the central axis increases. This yields a decrease of the azimuthal velocities near the central axis, which in turn decreases the pressure gradients in this region. Hence the sub-pressure near the nozzle decreases as can be seen by comparing Fig. 7 with 9. For the high swirling jet the largest pressure gradients are situated inside the jet, close to the nozzle and not further downstream around the central axis as was the case for the intermediate swirling jet. A decomposition of the pressure field in *P*_0_ and *P_S_* as in Fig. 10 shows the former is negligible compared to the latter. For high swirling jets, the radial momentum equation is dominated by the centrifugal forces and the simple radial equilibrium equation, Eq. 14, is valid.

### 4.5. Conclusions

In this paper the static pressure field of an annular swirling jet is measured indirectly using velocity field measurements. The pressure is computed by numerically solving the Poisson equation with the appropriate boundary conditions. The source terms of the Poisson equation are measured using stereoscopic-PIV. The advantages of this indirect method compared to the direct measurement by pitot tubes are an increased spatial resolution and the non-intrusive character of the technique. Four different swirl cases were investigated: a non-swirling, a low, an intermediate and a high swirling jet. For a low swirling jet, the static pressure field is the superposition of the pressure field of a non-swirling jet and a swirl induced pressure field. This swirl induced pressure field originates from the creation of radial pressure gradients to balance the centrifugal forces of the rotating jet. As the swirl is increased, the swirl induced pressure field dominates and the radial momentum equation can be simplified to the simple radial equilibrium equation, which expresses the balance between pressure gradients in the radial direction and centrifugal forces.

## Figures and Tables

**Figure 9.**
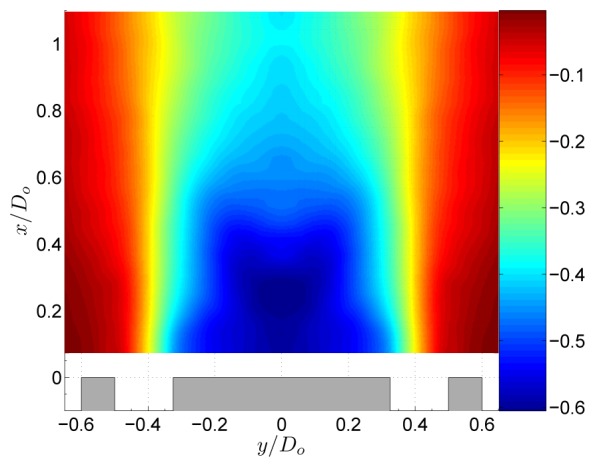
Normalised static pressure $P/\left(\rho U_{0}^{2}\right)$ of the annular jet at *S* = 0.74.

## References

[1] van Oudheusden B., Scarano F., Roosenboom E., Casimiri E., Souverein L. (2007). Evaluation of integral forces and pressure fields from planar velocimetry data for incompressible flows. Exp. Fluids.

[2] de Kat R., van Oudheusden B., Scarano F. Instantaneous planar pressure field determination around a square-section cylinder based on time-resolved stereo-piv.

[3] Gurka R., Liberzon A., Hefetz D., Rubinstein D., Shavit U. (1999). Computation of pressure distribution using piv velocity data. 3rd Int. Workshop on PIV.

[4] Leuckel W. (1969). Swirl intensities, swirl types and energy losses of diffe-rent swirl generating devices.

[5] Vanierschot M., Van den Bulck E. (2008). Influence of swirl on the initial merging zone of a turbulent annular jet. Phys. Fluids.

[6] Beér J., Chigier N., Robert E. (1983). Combustion Aerodynamics..

[7] Raffel M., Willert C., Kompenhans J. (1998). Partical Image Velocimetry, A practical Guide.

[8] Westerweel J. (1994). Efficient detection of spurious vectors in particle image velocimetry data. Exp. Fluids.

[9] Willert C. (1997). Stereoscopic digital particle image velocimetry for application in wind tunnel flows. Meas. Sci. Technol..

[10] Bendat J., Piersol A. (2000). Random Data: Analysis and Measurement Procedures..

[11] Ko N., Chan W. (1979). The inner regions of annular jets. J. Fluid Mech..

[12] Chigier N., Beer J. (1964). The flow region near the nozzle in double concentric jets. Trans. ASME, J. Basic Engng. D.

[13] Jeong J., Hussain F. (1995). On the identification of a vortex. J. Fluid Mech..

[14] Lucca-Negro O., O'Doherty T. (2001). Vortex breakdown: a review. Prog. Energy Combust. Sci..

[15] Gupta A. K., Lilley D. G., Syred N. (1984). Swirl Flows..

